# Supplementary material for: p53 Dysregulation in Breast Cancer: Insights on Mutations in the TP53 Network and p53 Isoform Expression
Source: Int J Mol Sci. 2023 Jun 13;24(12):10078. doi: 10.3390/ijms241210078 (PMC10298268; doi:10.3390/ijms241210078)
Supplement: Supplementary file 1 [file ijms-24-10078-s001.zip › Supplementary.pdf]

Supplementary

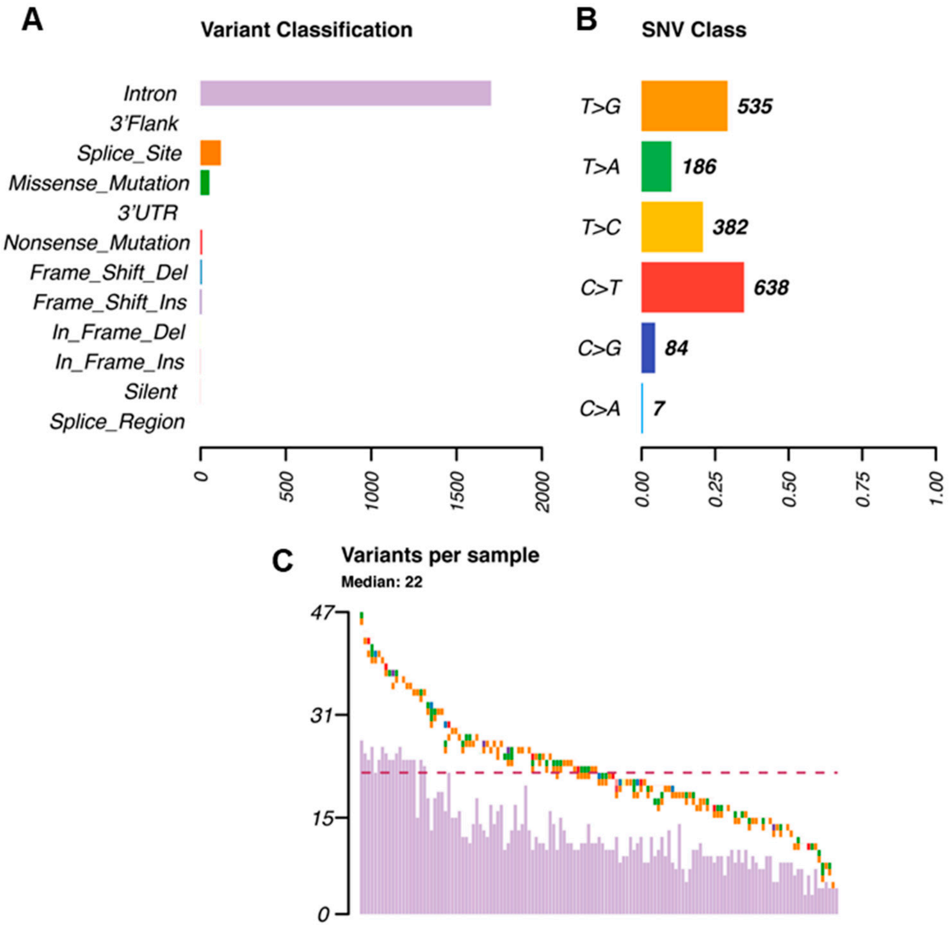

Suppl. Figure S1. Intronic variants are the most predominant variant type in IDCs. (**A**) Classification of *TP53* and (**B**) frequency of different SNV classes detected in 137 IDCs. (**C**) Representation of the number of *TP53* variants per sample. Alterations were detected in 100% of samples.

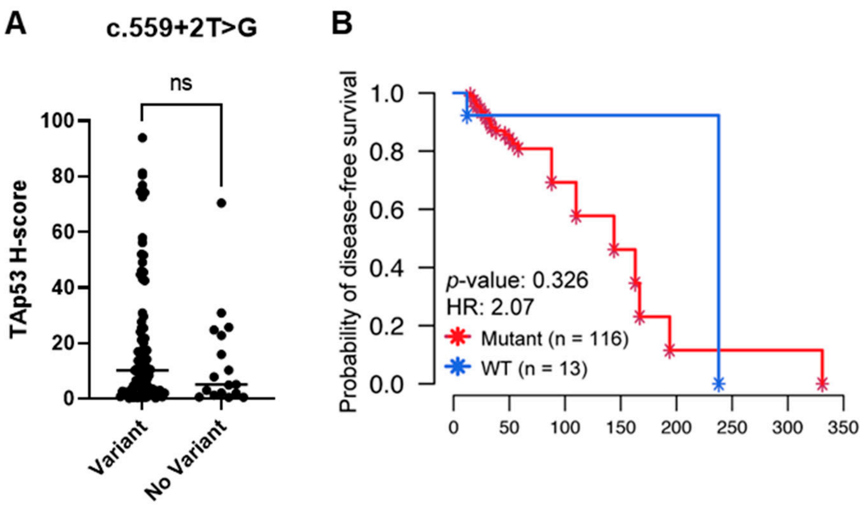

Suppl. Figure S2. The intronic substitution at a splice site c.559+2T>G of *TP53* is not associated with p53 expression or disease-free survival in IDCs. (**A**) TAp53 H-scores of 108 IDCs from our previous study [1] were segregated into presence (Variant) or absence (No Variant) of c.559+2T>G variant. (**B**) Kaplan–Meier survival curve representing disease-free survival of cases distributed based on *TP53* mutation status (including the c.559+2T>G variant) into wild-type (WT) or mutated. Mann-Whitney test was used to determine the statistical significance of c.559+2T>G variant and TAp53 levels. Log-rank (Mantel–Cox) test was used to determine the statistical significance of *TP53* mutation status and disease-free survival. Results were considered significant at $p < 0.05$.

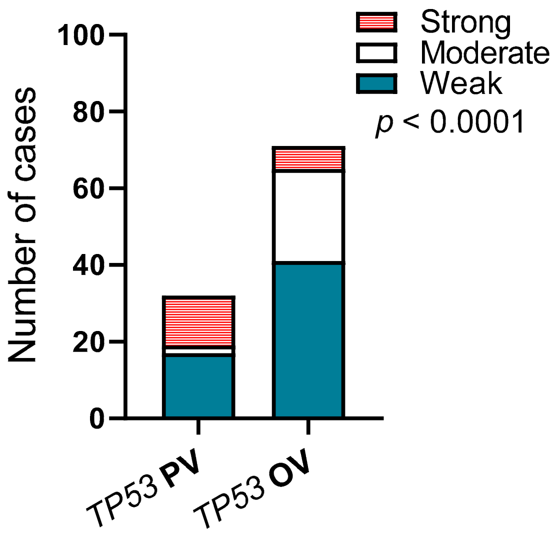

Suppl. Figure S3. *TP53* pathogenic variants are enriched in strong and weak TAp53-expressing IDCs. *TP53* mutations were separated into pathogenic variants (PV) and other variants (OV). TAp53 H-scores of 108 IDCs from our previous study [1] were segregated into weak, moderate, and high levels [2]. Statistical analysis was performed using Pearson's chi-square test. Results were considered significant at $p < 0.05$.

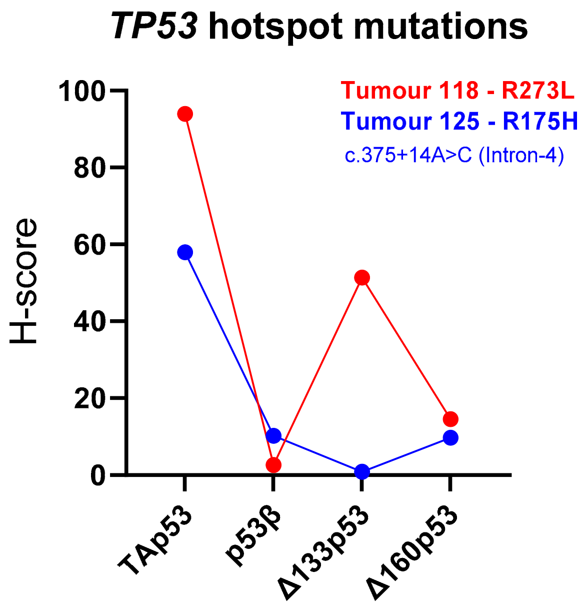

Suppl. Figure S4. p53 isoform expression in samples harbouring *TP53* hotspot mutations. p53 isoform H-scores of two IDCs from our previous study [1], which harboured *TP53* hotspot mutations: R273L (c.818G>T) and R175H (c.524G>A). Tumour 125 also harboured a sequence variant (c.375+14A>C) in intron 4.

References

1. Steffens Reinhardt, L.; Groen, K.; Morten, B. C.; Bourdon, J.-C.; Avery-Kiejda, K. A., Cytoplasmic p53β Isoforms Are Associated with Worse Disease-Free Survival in Breast Cancer. *International Journal of Molecular Sciences* **2022,** *23* (12), 6670.
2. Bankhead, P.; Fernández, J. A.; McArt, D. G.; Boyle, D. P.; Li, G.; Loughrey, M. B.; Irwin, G. W.; Harkin, D. P.; James, J. A.; McQuaid, S.; Salto-Tellez, M.; Hamilton, P. W., Integrated tumor identification and automated scoring minimizes pathologist involvement and provides new insights to key biomarkers in breast cancer. *Laboratory Investigation* **2018,** *98* (1), 15-26.
